# Epidemiology of Plasmids in *Escherichia coli* and *Klebsiella pneumoniae* with Acquired Extended Spectrum Beta-Lactamase Genes Isolated from Chronic Wounds in Ghana

**DOI:** 10.3390/antibiotics11050689

**Published:** 2022-05-19

**Authors:** Frederik Pankok, Stefan Taudien, Denise Dekker, Thorsten Thye, Kwabena Oppong, Charity Wiafe Akenten, Maike Lamshöft, Anna Jaeger, Martin Kaase, Simone Scheithauer, Konstantin Tanida, Hagen Frickmann, Jürgen May, Ulrike Loderstädt

**Affiliations:** 1 Institute for Infection Control and Infectious Diseases, University Medical Center Göttingen, 37075 Göttingen, Germany; frederik.pankok@med.uni-goettingen.de (F.P.); stefan.taudien@med.uni-goettingen.de (S.T.); martin.kaase@med.uni-goettingen.de (M.K.); simone.scheithauer@med.uni-goettingen.de (S.S.); 2 Department of Implementation Research, Bernhard Nocht Institute for Tropical Medicine Hamburg, 20359 Hamburg, Germany; dekker@bnitm.de; 3 Department of Infectious Disease Epidemiology, Bernhard Nocht Institute for Tropical Medicine Hamburg, 20359 Hamburg, Germany; thye@bnitm.de (T.T.); lamshoeft@bnitm.de (M.L.); anna.jaeger@bnitm.de (A.J.); may@bnitm.de (J.M.); 4 Kumasi Centre for Collaborative Research in Tropical Medicine (KCCR), Kumasi 039-5028, Ghana; Oppong.kwabena@presbyuniversity.edu.gh (K.O.); danquah@kccr.de (C.W.A.); 5 German Center for Infection Research (DZIF), Partner Site Hamburg-Lübeck-Borstel-Riems, 80331 Munich, Germany; 6 Department of Microbiology and Hospital Hygiene, Bundeswehr Hospital Hamburg, External Site at the Bernhard Nocht Institute for Tropical Medicine Hamburg, 20359 Hamburg, Germany; Konstantin.tanida@gmail.com (K.T.); frickmann@bnitm.de (H.F.); 7 Institute for Medical Microbiology, Virology and Hygiene, University Medicine Rostock, 18057 Rostock, Germany; 8 Tropical Medicine II, University Medical Center Hamburg-Eppendorf (UKE), 20251 Hamburg, Germany

**Keywords:** chronic wound infection, *Klebsiella pneumoniae*, *Escherichia coli*, plasmid, resistance genes, mobile genetic element, Enterobacterales, Ghana, phylogeny

## Abstract

Little information is available on the local epidemiology of mobile genetic elements such as plasmids harboring acquired beta-lactamase genes in Western African Ghana. In the present study, we screened for plasmids in three *Escherichia coli* and four *Klebsiella pneumoniae* isolates expressing extended spectrum beta-lactamases (ESBL) mediated by the *bla*_CTX-M-15_ gene from chronically infected wounds of Ghanaian patients. Bacterial isolates were subjected to combined short-read and long-read sequencing to obtain the sequences of their respective plasmids. In the *bla*_CTX-M-15_-gene-carrying plasmids of the four ESBL-positive *K. pneumoniae* isolates, IncFIB/IncFII (*n* = 3) and FIA (*n* = 1) sequences were detected, while in the *bla*_CTX-M-15_-gene-carrying plasmids of the three ESBL-positive *E. coli* isolates, IncFIA/IncFIB (*n* = 2) and IncFIB (*n* = 1) sequences were found. The three IncFIB/IncFII sequence-containing plasmids were almost identical to a *K. pneumoniae* plasmid reported from France. They belonged to the clonal lineages ST17, ST36 and ST39 of *K. pneumoniae*, suggesting transversal spread of this obviously evolutionary successful plasmid in Ghana. Other resistance gene-encoding plasmids observed in the assessed Enterobacterales harbored IncFIA/IncR and IncFII sequences. International spread was confirmed by the high genetic similarity to resistance-mediating plasmids published from Asia, Australia, Europe and Northern America, including a *bla*_CTX-M-15_-gene-carrying plasmid isolated from a wild bird in Germany. In conclusion, the study contributed to the scarcely available information on the epidemiology of third-generation cephalosporine resistance-mediating plasmids in Ghana. Furthermore, the global spread of resistance-mediating plasmids provided hints on the evolutionary success of individual resistance-harboring plasmids by transversal spread among *K. pneumoniae* lineages in Ghana.

## 1. Introduction

In recent years, multidrug resistance has become a major concern in sub-Saharan Africa. It has made bacterial infections increasingly difficult to treat, especially those associated with Gram-negative pathogens [1,2,3,4,5,6]. Acquired antimicrobial drug resistance in Gram-negative bacteria is typically mediated by mobile genetic elements such as plasmids, whose horizontal spread is driven by conjugation-based transmission [7]. Their persistence in bacterial clones is influenced by both fitness costs for the bacterial hosts [8] as well as by compensatory mutations [7,9]. The latter comprise, e.g., mutations in intergenic regions and the selection of genes involved in anaerobic metabolism [10].

In Enterobacterales such as *Escherichia coli* and *Klebsiella pneumoniae*, multidrug resistance is frequently mediated by epidemic resistance plasmids of incompatibility (Inc) groups such as IncFII, IncA/C, IncL/M, IncN and IncI1, which carry genes for extended-spectrum beta-lactamases (ESBLs), AmpC beta-lactamases and carbapenemases [11,12,13,14,15,16,17,18,19,20,21,22,23,24,25,26,27,28,29,30,31,32,33,34,35,36,37,38]. Next-generation sequencing-based approaches have been introduced early for the identification of plasmid sequences [39,40,41].

Epidemiological information on the spread and distribution of resistance-mediating plasmids in bacteria in Ghana is scarce besides individual approaches such as a study on the diversity of plasmids in Ghanaian gonococci from the beginning of the 1980s [42]. Regional spread of a trimethoprim resistance gene cassette via a successful transposable element was reported for *Escherichia coli* strains isolated in Ghana between 2006 and 2008 [43]. The conjugation-based transfer of *bla*_TEM_-gene-mediated ESBL expression could be shown for two-thirds of *bla*_TEM_ gene-positive Enterobacterales isolated at a Ghanaian tertiary hospital [44]. In Ghanaian salmonellae comprising ESBL-positive strains mediated by the beta-lactamase genes *bla*_TEM52-B_ and *bla*_CTX-M15_, IncN-type, IncFII(S)/IncFIB(S)/IncQ1-type, IncX1-type and TrfA/IncHI2/IncHI2A-type plasmids have been reported [45]. In an *Escherichia coli* isolate of the ST410 sequence type, the IncHI-type transferrable plasmid p2189-NDM was described, carrying the resistance genes *bla*_NDM-1_, *bla*_CTX-M-15_, *aadA1*, *aac(6’)-Ib*, *sul3*, *dfrA12* and *cmlA1* [46]. In *Klebsiella pneumoniae* isolates from a teaching hospital, IncFIB(K)-type and ColRNAI-type plasmids harbored resistance genes such as *bla*_CTX-M-15_, *bla*_SHV-11_, *bla*_TEM-1B_, *bla*_OXA-1_, *ac(3)-IIa*, *strB*, *strA*, *aadA16*, *qnrB66*, *oqxA* and *oqxB*, [47]. Various other epidemiological studies conducted in Ghana provide information on locally abundant resistance mechanisms without further addressing transposable genetic elements [48,49,50,51,52,53].

Recently, a predominance of Gram-negative rod-shaped bacteria was identified in chronic wounds in rural Ghana [54] with only low to moderate resistance rates compared to other reports from Ghanaian hospitals [55]. Among the Enterobacterales, a minority of three *E. coli* and four *K. pneumoniae* expressing the *bla*_CTX-M-15_ gene with a resulting ESBL-phenotype [56] were identified.

In the present study, the mobile genetic elements within those ESBL-producing Enterobacterales from chronic wounds of Ghanaian patients were assessed. By doing so, the so far scarce knowledge on the local epidemiology of plasmids mediating acquired antimicrobial resistance in Enterobacterales from Ghana was investigated.

## 2. Results

**Figure 1:**
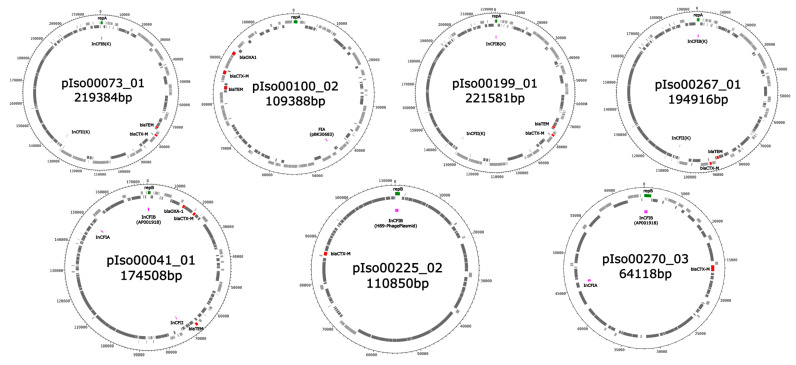
Visualization of the *bla*_CTX-M_-gene-carrying plasmids. Upper row: Plasmids detected in *K. pneumoniae*. Lower row: Plasmids detected in *E. coli*. Genes located on the forward and reverse strand are colored in light and dark grey, respectively. *Bla* genes are shown in red. Genes coding for replication-associated proteins and marking the start gene of the sequence are shown in green. PlasmidFinder matches are shown in purple.

From seven Enterobacterales isolates from chronic wounds in Ghana as characterized in the methods chapter, 28 plasmid sequences were detected in four assessed ESBL-positive *K. pneumoniae* strains and in three *E. coli* strains. The sizes of the recorded plasmid contigs ranged from 1538 to 224,675 base pairs (Table 1). GenBank accession numbers and typing results applying the software Plasmidfinder 2.0 and mob-typer are shown in Table 1. The most frequently detected PlasmidFinder 2.0 and mob-typer matches for Inc sequences comprised IncFII (*n* = 5), IncFIA (*n* = 4), IncFIB (*n* = 4), IncFIC (*n* = 1), IncP1 (*n* = 1) and IncR (*n* = 1) sequences in 9 out of 16 plasmid sequences from ESBL-positive *K. pneumoniae* isolates. The ESBL-encoding *bla*_CTX-M-15_ genes were carried on plasmids of the IncFIB/IncFII type in three out of four *K. pneumoniae* strains and in another instance on an IncFIA type plasmid. In the plasmids from the three ESBL-positive *E. coli*, IncFIB (*n* = 4), IncFIA (*n* = 2), IncFII (*n* = 1), IncFIC (*n* = 1) and IncY (*n* = 1) sequences were detected in 7 out of 12 plasmid sequences. The ESBL-encoding *bla*_CTX-M-15_ genes were located on plasmids of the IncFIA/IncFIB/IncFIC-like type, the IncFIA/IncFIB/IncFII type and the IncFIB-type in *E. coli* strains. The *bla*_CTX-M-15_-carrying plasmids of both *K. pneumoniae* and *E. coli* strains are visualized in Figure 1. As suggested by the mob-typer software, predicted mobilities of the plasmids comprised the following categories: conjugative (*n* = 8, including 4 *bla*_CTX-M-15_ gene harboring plasmids), mobilizable (*n* = 11, including 2 *bla*_CTX-M-15_ gene harboring plasmids) and non-mobilizable (*n* = 9, including 1 *bla*_CTX-M-15_ gene harboring plasmid) (Table 1).

Resistance genes were located on 8 out of 16 plasmids of the four ESBL-positive *K. pneumoniae* isolates, as well as on 4 out of 12 plasmids of the three ESBL-positive *E. coli* isolates, as detailed in Table 1. In three out of four instances in *K. pneumoniae*, the *bla*_CTX-M-15_ gene was associated with the aminoglycoside-mediating gene *aac(3)-IIa* and the narrow-spectrum beta-lactamase gene *bla*_TEM-1B_ on the same plasmid. One *bla*_CTX-M-15_ gene harboring plasmid in *K. pneumoniae* was associated with multiple resistance genes comprising macrolide resistance-mediating *mph(A)*; aminoglycoside resistance-mediating *aph(3′)-Ia aph(3″)-Ib*, *aph(6)-Id*, *aac(3)-IIa* and *aadA16*; sulfonamide resistance-mediating *sul1* and *sul2*; disinfectant resistance-mediating *qacE*; quinolone resistance-mediating *qnrB6*; trimethoprim-resistance-mediating *dfrA27*; rifampicin resistance-mediating *ARR-3*; fluoroquinolone and aminoglycoside-resistance-mediating *aac(6′)-Ib-cr*; the narrow-spectrum beta-lactamases *bla*_TEM-1B_ and *bla*_OXA-1_; and phenicol resistance-mediating *catB3*. In the three ESBL-positive *E. coli* strains, the *bla*_CTX-M-15_ gene was the only resistance gene located on the plasmid in one instance. In another instance, it was associated with phenicol resistance-mediating *catA1* and tetracycline resistance-mediating *tet(B)*. In the third strain, an association with phenicol resistance-mediating *catA1* and *catB3*, the narrow-spectrum beta-lactamases *bla*_OXA-1_ and *bla*_TEM-1B_, fluoroquinolone and aminoglycoside resistance-mediating *aac(6′)-Ib-cr*, disinfectant resistance-mediating *sitABCD* and *qacE*, tetracycline resistance-mediating *tet(B)*, macrolide resistance-mediating *mph(A)*, sulfonamide resistance-mediating *sul1*, aminoglycoside resistance-mediating *aadA5* and *aac(3)-IId,* as well as trimethoprim resistance-mediating *dfrA17,* was recorded.

Of note, three out of four *bla*_CTX-M-15_-gene-carrying plasmids from *K. pneumoniae* isolates of different clonal lineages showed a very high genetic similarity to a plasmid isolated from a *K. pneumoniae* isolate from France (Appendix A, Table A1 and Table A2). This similarity was confirmed by a BlastN comparison of all *bla*_CTX-M-15_ gene harboring plasmids from the present study, confirming the high genetic similarity of the plasmids pIso00073_01, pIso00199_01 and pIso00267_01 from the *K. pneumoniae* clonal complex isolates ST39, ST17 and ST36, respectively (Table 2). In the mob-typer analysis, these plasmids were characterized as conjugative (Table 1).

As indicated by another BlastN search (Appendix A), similar resistance-carrying plasmids to the ones from the present study have been globally isolated and sequenced in Europe, Asia, Australia and North America. The international isolations were performed not only from human samples but also from a sample taken from a wild bird, as reported from Germany (Appendix A).

## 3. Discussion

The study was conducted to add epidemiological information on the local epidemiology of third-generation cephalosporine resistance-mediating plasmids in Enterobacterales isolated from chronic wounds in Ghana. To do so, four *K. pneumoniae* and three *E. coli* strains carrying the ESBL-mediating *bla*_CTX__-M-15_ genes were chosen from a previous study [54,56]. Wound isolates were chosen due to their likely etiological relevance for human infections, although etiologically irrelevant colonization cannot be completely ruled out at primarily non-sterile sampling sites such as superficial wounds in contact with the environment. Phenotypical strain characteristics did not affect the choice, which included all isolated *bla*_CTX-M-15_-gene-carrying Enterobacterales from the previous assessment. The strains were subjected to combined long-read and short-read sequencing to identify resistance-encoding plasmids and to compare the results with previous assessments.

In concordance with our results, IncFIB-type plasmids found in *K. pneumoniae* isolates from Ghana associated with the *bla*_CTX__-M-15_ gene had been previously described in 2019 by Agyepong and colleagues [47]. In addition to the previously published results, we detected three plasmids with IncFIB/IncFII sequences that had previously been reported from France (GenBank accession number LR991402.1). Very similar, although not completely identical, plasmids were found in Ghanaian *K. pneumoniae* strains of the clonal lineages ST17, ST36 and ST39, suggesting the horizontal transmission of this plasmid within *K. pneumoniae* strains in Ghana, as also confirmed by mobility prediction with the mob-typer software. Of note, ESBL-positive ST39 *K. pneumoniae* strains have previously been reported to be highly prevalent in pigs and abattoir workers in Cameroon [57]. A *bla*_CTX__-M-15_-gene-carrying plasmid of the FIA type found in another *K. pneumoniae* strain was previously described by Canadian scientists (GenBank accession number CP023950.1), confirming its international spread. Plasmids of the incompatibility groups IncFIA, IncFIB and IncFII carrying *bla*_CTXM-15_ genes have also been described from Eastern African Tanzania [58]. In Tanzanian *K. pneumoniae* strains, in particular, a *bla*_CTXM-15_-gene-harboring plasmid of the incompatibility group IncFIIK5/IncR has been associated with highly efficient horizontal transfer [59].

The description of plasmids carrying IncFIA/IncFIB/IncFIC, IncFIA/IncFIB/IncFII and IncFIB sequences associated with *bla*_CTX-M-15_ genes in *E. coli* strains is new and adds to the available information on the epidemiology of plasmids encoding resistance against third-generation cephalosporines in Gram-negative pathogens in Ghana [46,47]. Interestingly, genetically highly similar plasmid sequences have been reported from Australia (GenBank accession number LR890289.1), the United Kingdom (UK) [60] and Germany [61] before, comprising a human *E. coli* isolate from the UK and an *E. coli* strain isolated from a wild bird in Germany. It has recently been suggested [62] that not only international travel but also bacterial spread by migrating birds might contribute to the distribution of resistant bacterial isolates and their resistance-encoding plasmids. A single isolation from a bird cannot be considered as definitive proof because contamination from human sources remains an option but is nevertheless in line with this hypothesis. Interestingly, plasmids of the incompatibility group IncY have been linked to *bla*_CTXM-15_ gene-carriage in Tanzanian *E. coli* strains [63], while an IncY plasmid from one of the assessed Ghanaian *E. coli* strains did not harbor any resistance-associated genes.

Resistance against several antibiotic drugs other than beta-lactams, which had been phenotypically observed for the assessed Ghanaian Enterobacterales, was shown to be caused by co-occurring plasmids. In *K. pneumoniae*, such plasmids comprised the types IncFIA, IncFIA/IncR, IncFIA/IncFIC/IncFII and IncFIB/IncFII, while in *E. coli*, an IncFIA/IncFIB/IncFII-type plasmid encoded multiple resistance genes. Resistance-gene-carrying IncFIC plasmids in Africa have also been described in multidrug-resistant *Salmonella enterica* isolated in Kenya [64]. In contrast, ColRNAI-type plasmids, which were associated with antimicrobial resistance in Ghanaian *K. pneumoniae* strains in a previous study [47], did not encode resistance genes in our assessment.

As reported previously [39], linking of different contigs on the same plasmid can be challenging due to technical limitations of the sequencing technology. With focus on this technical issue, double sequencing with short-read (Illumina) and long-read (Nanopore) technologies was performed, followed by hybrid assembly of both data sets. Furthermore, evidence of plasmid replicon sequences was secured using three different methods (PlasmidFinder, MOB typing and BlastN versus the SRST2 database [65]) for all contigs. Furthermore, the plasmid nature of these shorter contigs is supported by normalized depths from 1.3 to 12.1 with an average of 4.2, as calculated by the Unicyler assembler (chromosome set to 1.0). Admittedly, these procedures do not provide 100% proof but were considered as sufficient justification for reporting the contigs as plasmids.

The study has a few limitations. First and most important, the number of ESBL-positive isolates available from the previous study [56] for inclusion in the plasmid assessment was considerably low. Accordingly, a regional representativeness cannot be ensured, although partial matching with previously published results from Ghana could be demonstrated [47]. Second, although the inclusion of etiologically relevant strains was aspired to by including strains from a wound infection study [54,56] instead of screening isolates, etiological relevance of the included strains is not definitely assured because the discrimination of causative infectious agents and colonizing bacterial flora is challenging at primarily non-sterile sites such as chronic wounds. Third, the postulated horizontal spread of plasmid-mediated third-generation cephalosporine resistance in Ghana was not confirmed by conjugation experiments. Such approaches would have been beyond both the scope and the financial options of this investigator-initiated, solely epidemiological study.

## 4. Materials and Methods

### 4.1. Sample Collection, Bacterial Culture, Antibiotic Susceptibility Testing and Whole Genome Sequencing

A total of seven ESBL-positive Enterobacterales carrying the *bla*_CTX-M-15_ gene were selected from a previous study on bacterial isolates from chronic wounds of patients from rural Ghana [54,56]. In summary, the strains were isolated from patients ≥ 15 years with infected chronic wounds at the Outpatient Department (OPD) of the Agogo Presbyterian Hospital in the Asante Akim North District of rural Ghana. Antibiotic resistance, as indicated in the Appendix A below, was assessed by the disk diffusion method and interpreted following the European Committee on Antimicrobial Susceptibility Testing (EUCAST) guidelines v.6.0 (http://www.eucast.org (accessed on 31 January 2016)). Both species identity and antibiotic susceptibility had been confirmed using the VITEK2 system (bioMérieux, Nürtingen, Germany), as described elsewhere [54,56].

Following nucleic acid extraction applying the MasterPure Complete DNA and RNA Purification Kit (LGC standards GmbH, Wesel, Germany), DNA was sent for whole genome sequencing (WGS) to BGI Europe, Denmark, Copenhagen. There, a BGISEQ-500 device was used for sequencing, generating 2 × 150 bp paired-end reads with an aimed coverage of 100×. Short-read archive (SRA) accession numbers of the obtained sequences are indicated in Table 1, linking to the original raw data as uploaded for public use to the short-read archive (SRA, NCBI) under the accession number PRJNA699140 [56]. Details on the chosen isolates are provided in Appendix A.

In addition, 1 µg DNA was sent to BGI Genomics C, Ltd., for long-read sequencing. In detail, sequencing analysis was performed on a PormethION (Nanopore) device using the flow-cell version R.9.4.1. Base-calling was performed with the Guppy software (https://nanoporetech.com, last accessed on 19 May 2022) applying the high-accuracy (HAC) model. After sequencing, about 3 mb of data was obtained from each sample.

### 4.2. Assessment of the Whole Genome Sequencing Data for Plasmids

Assembly of long-read sequences by Flye, v2.9 (https://github.com/fenderglass/Flye, last accessed on 30 March 2022) using (i) unfiltered fastq reads, (ii) fastq reads > 3 kb and (iii) fastq reads > 3 kb with subsequent polishing by Medaka v1.5.0 (https://github.com/nanoporetech/medaka, last accessed on 30 March 2022) did not reveal significant differences with respect to the assembled contigs. However, as expected, hybrid assemblies of long- and short-read sequences were of remarkably higher quality due to the resolution of sequencing errors in homo-nucleotide stretches (data not shown), a shortcoming of the nanopore technology [66]. Therefore, hybrid assemblies were performed by Unicycler [67] using unfiltered Oxford nanopore long-reads and BGI paired-end short-reads (after fastqc, trimmomatic). This resulted in assemblies containing one chromosome contig (*K. pneumoniae* 5.3–5.4 Mb; *E. coli* 4.7–5.0 Mb) and up to six plasmids per genome. Furthermore, the assembled plasmid contigs were analyzed by blastN (https://blast.ncbi.nlm.nih.gov/Blast.cgi, last accessed on 30 March 2022) vs. “Bacteria (taxid:2)” [68]. Results were filtered for the highest query coverage (as shown in Appendix A). Annotation was conducted by RAST (http://rast.nmpdr.org, last accessed on 30 March 2022). Analysis of the assembled genomes for resistance genes was conducted by ResFinder (http://cge.cbs.dtu.dk/services/ResFinder/—version 4.1, last accessed on 30 March 2022), with a nucleotide identity threshold of 90% and a minimum match length of 60%. Detection of plasmids was performed by PlasmidFinder, version 2.0.1 (https://cge.cbs.dtu.dk/services/PlasmidFinder-2.0/, last accessed on 30 March 2022) [39,69] and by the mob-typer software (https://github.com/phac-nml/mob-suite, last accessed on 14 April 2022) to identify matching incompatibility types [70] and to predict the mobility of the plasmids. As a third procedure, BlastN versus the SRST2 database [65] was conducted to ensure evidence of plasmid replicon sequences for all contigs, which were named IsoXXXXX_pXX. The sizes of the plasmids were based on the Unicycler results (short- and long-read hybrid assembly), which represents the most reliable approach currently available. Regarding the position of the *bla*_CTX-M-15_ genes within the plasmid sequences, the ResFinder results were independently confirmed by PROKKA annotation and Abricate [71] analysis, using the NCBI AMR Finder Plus Database, of the contigs of the above-mentioned hybrid assemblies (data not shown [72]). The ResFinder output provided the coordinates of the detected genes within the analyzed nucleotide sequences. For visualization purposes, the plasmid nucleotide sequences were annotated via RASTk using default settings [73]. The generated merged GenBank files were visualized using the tool DNAplotter [74].

### 4.3. Ethical Clearance

Ethical clearance was provided by the Committee on Human Research, Publications and Ethics, School of Medical Science, Kwame Nkrumah University of Science and Technology in Kumasi, Ghana (approval number CHRPE/AP/078/16).

## 5. Conclusions

Despite the above-mentioned limitations, which narrow the interpretability of the results, the study adds to the scarcely available data on the epidemiology of plasmids encoding third-generation cephalosporine resistance in ESBL-positive Enterobacterales in Western African Ghana. At least for *K. pneumoniae*, an individual conjugative *bla*_CTX-M-15_-gene-carrying plasmid was found in three out of four assessed strains of different clonal lineages, suggesting successful vertical transmission in Ghana. Furthermore, the assessment exemplarily demonstrated the international spread of such resistance-mediating plasmids in times of globalization, affected, e.g., by human travelling and the migration of wild birds. Future assessments should comprise more sampling sites, clinical conditions and geographic locations in Ghana to provide more robust and conclusive epidemiological data compared to the present hypothesis-forming study.

## Figures and Tables

**Table 1:** Identified plasmids with information on size, typing results based on PlasmidFinder-2.0 and mob-typer, predicted mobility based on mob-typer and encoded resistance genes. Resistance genes occurring with more than one copy are marked with (*).

Species and Isolate Number/MLST Type of the Isolate/GenBank Accession Number of Genomic DNA	Plasmid Id and GenBank Accession Number	Plasmid Size	Typing Results Based on PlasmidFinder-2.0 and Mob-Typer	Predicted Mobility Based on Mob-Typer	Resistance Genes on the Plasmid
*K. pneumoniae* Iso00073/ST39/CP095150	pIso00073_01; CP095151	219,384	IncFIB, IncFII, rep_cluster_2183	conjugative	*aac(3)-IIa*, *bla*_TEM-1B_ *, *bla*_CTX-M-15_
	pIso00073_02; CP095152	92,025	IncFIA, IncR	mobilizable	*sul2*, *aph(3″)-Ib*, *aph(3″)-Ib*, *aph(6)-Id*, *bla*_TEM-1B_ *, *catA2*-like, *tet(D)*, *aac(6′)-Ib-cr*, *ARR-3*, *dfrA27*, *aadA16*, *qacE*, *sul1*, *qnrB2*
	pIso00073_03; CP095153	82,442	IncFII, IncFIA, IncFIC	conjugative	*erm(B)*, *mph(A)*, *bla*_TEM-1B_ *
	pIso00073_04; CP095154	4350	ColRNAI_rep_cluster_1987	non-mobilizable	-
*K. pneumoniae* Iso00100/ST152/CP095145	pIso00100_01; CP095146	224,675	IncFIB, IncFII, rep_cluster_2183	conjugative	*dfrA1*, *aadA1*, *qacE*, *sul1*, tet(D), *bla*_SHV-187_ *, *catA1*
	pIso00100_02; CP095147	109,388	FIA, rep_cluster_1418	mobilizable	*mph(A)*, *aph(3’)-Ia*, *sul1*, *qacE* *, *qnrB6*, *aadA16*, *dfrA27*, *ARR-3*, *aac(6’)-Ib-cr* *, *sul2*, *aph(3’’)-Ib*, *aph(6)-Id*, *bla*_TEM-1B_, *bla*_CTX-M-15_, *bla*_OXA-1_, *catB3*, *aac(3)-IIa*
	pIso00100_03; CP095148	8282	ColRNAI_rep_cluster_1857	mobilizable	-
	pIso00100_04; CP095149	4642	Col440I, ColRNAI_rep_cluster_1987	non-mobilizable	-
*K. pneumoniae* Iso00199/ST17/CP095140	pIso00199_01; CP095141	221,581	IncFIB, IncFII, rep_cluster_2183	conjugative	*aac(3)-IIa*, *bla*_TEM-1B_ *, *bla*_CTX-M-15_
	pIso00199_02; CP095142	52,096	IncP1	conjugative	-
	pIso00199_03; CP095143	4204	rep_cluster_2358	non-mobilizable	-
	pIso00199_04; CP095144	3643	ColRNAI_rep_cluster_1987	non-mobilizable	-
*K. pneumoniae* Iso00267/ST36/CP095132	pIso00267_01; CP095133	194,916	IncFIB, IncFII, rep_cluster_2183	conjugative	*aac(3)-IIa*, *bla*_TEM-1B_, *bla*_CTX-M-15_
	pIso00267_02; CP095134	70,936	IncFIA, rep_cluster_1418	mobilizable	*tet(D)*, *catA2*-like, *aph(6)-Id* *, *aph(3’’)-Ib* *, *sul2*, *aac(6’)-Ib-cr*, *ARR-3*, *dfrA27*, *aadA16*, *qacE*, *sul1*
	pIso00267_03; CP095135	9294	ColRNAI_rep_cluster_1857	mobilizable	-
	pIso00267_04; CP095136	5835	Col(Ye4449)	mobilizable	-
*E. coli* Iso00041/ST2/CP095155	pIso00041_01; CP095156	174,508	IncFIA, IncFIB, IncFIC	conjugative	*catB3*, *bla*_OXA-1_, *aac(6′)-Ib-cr*, *sitABCD*, *bla*_CTX-M-15_, *tet(B)*, *catA1*, *mph(A)*, *sul1*, *qacE*, *aadA5*, *dfrA17*, *aac(3)-IId*, *bla*_TEM-1B_
	pIso00041_02; CP095157	5164	IncFIB, rep_cluster_2131	mobilizable	-
	pIso00041_03; CP095158	2348	IncFII, Col(IRGK)	non-mobilizable	-
	pIso00041_04; CP095159	1538	Col(MG828)	mobilizable	-
*E. coli* Iso00225/ST506/CP095137	pIso00225_01; CP095138	133,313	Col156, IncFIA, IncFIB, IncFII, rep_cluster_2131	conjugative	*tet(A)*, *aph(6)-Id*, *aph(3″)-Ib*, *sul2*, *mph(A)*, *sul1*, *qacE*, *aadA5*, *dfrA17*
	pIso00225_02; CP095139	110,850	IncFIB, rep_cluster_488	non-mobilizable	*bla*_CTX-M-15_
*E. coli* Iso00270/ST2/CP095125	pIso00270_01; CP095126	99,933	IncY	non-mobilizable	-
	pIso00270_02; CP095127	94,223	rep_cluster_1704	non-mobilizable	-
	pIso00270_03; CP095128	64,118	IncFIA, IncFIB, IncFII	mobilizable	*catA1*, *bla*_CTX-M-15_, *tet(B)*
	pIso00270_04; CP095129	5164	rep_cluster_2131	mobilizable	-
	pIso00270_05; CP095130	3007	rep_cluster_2350	mobilizable	-
	pIso00270_06; CP095131	2255	Col(MG828)	non-mobilizable	-

**Table 2:** Sequence homology as assessed by pairwise blastN analysis of *bla*_CTX-M_-gene containing plasmids/query coverages.

			*Klebsiella pneumoniae*				*Escherichia coli*		
			Iso00073	Iso00199	Iso00267	Iso00100	Iso00041	Iso00225	Iso00270
			pIso00073_01	pIso00199_01	pIso00267_01	pIso00100_02	pIso00041_01	pIso00225_02	pIso00270_03
*Klebsiella pneumoniae*	Iso00073	pIso00073_01	-	100%	83%	9%	11%	1%	7%
	Iso00199	pIso00199_01	99%	-	83%	9%	11%	1%	7%
	Iso00267	pIso00267_01	92%	92%	-	12%	11%	1%	8%
	Iso00100	pIso00100_02	21%	21%	25%	-	35%	2%	14%
*Escherichia coli*	Iso00041	pIso00041_01	15%	15%	16%	19%	-	1%	27%
	Iso00225	pIso00225_02	2%	2%	2%	2%	2%	-	11%
	Iso00270	pIso00270_03	23%	23%	24%	18%	65%	3%	-

## Data Availability

All relevant data have been provided in the paper. Raw data are available via the links indicated in the paper and can also be provided by the authors on reasonable request.

## Appendix A

**Table A1:** Plasmids carrying antimicrobial resistance genes and disinfectant resistance genes and genetic similarity according to BlastN matching with previously published plasmids in the NCBI database, as well as the previous sites of detection.

Strain/Sequence Type (ST)/GenBank Accession Number of Genomic DNA	Plasmid Number; GenBank Accession Number	Best Hit with Respect to the Query Coverage	Query Coverage (%)	Nucleotide Identity (%)	Geographic Site of NCBI Sequence Submission (Bacterial Species and Source) [Reference]
*K. pneumoniae* Iso00073/ST39/CP095150	pIso00073_01; CP095151	LR991402.1	97%	99.99%	France (*K. pneumoniae*, source: unknown) [none]
	pIso00073_02; CP095152	CP063009.1	94%	99.99%	Russian Federation (*K. pneumoniae*, source: human) [75]
	pIso00073_03; CP095153	CP054171.1	95%	99.86%	India (*K. pneumoniae*, source: human) [none]
*K. pneumoniae* Iso00100/ST152/CP095145	pIso00100_01; CP095146	CP065826.1	79%	99.95%	United States of America, (*K. pneumoniae*, source: human) [none]
	pIso00100_02; CP095147	CP023950.1	80%	99.48%	Canada (*K. pneumoniae*, source: human) [none]
*K. pneumoniae* Iso00199/ST17/CP095140	pIso00199_01; CP095141	LR991402.1	96%	99.99%	France (*K. pneumoniae*, source: unknown) [none]
*K. pneumoniae* Iso00267/ST36/CP095132	pIso00267_01; CP095133	LR991402.1	94%	99.99%	France (*K. pneumoniae*, source: unknown) [none]
	pIso00267_02; CP095134	CP016810.1	72%	99.97%	United States of America, (*K. pneumoniae*, source: human) [none]
*E. coli* Iso00041/ST 2/CP095155	pIso00041_01; CP095156	LR890289.1	99%	99.98%	Australia (*E. coli*, source: unknown) [none]
*E. coli* Iso00225/ST506/CP095137	pIso00225_01; CP095138	CP088462.1	100%	100%	South Korea (*E. coli*, source: human) [none]
	pIso00225_02; CP095139	MW590712.1	100%	100%	United Kingdom (*E. coli*, source: human) [60]
*E. coli* Iso00270/ST2/CP095125	pIso00270_03; CP095128	CP023816.1	96%	99.11%	Germany (*E. coli*, source: wild bird) [61]

**Table A2:** Strain-specific details of the 7 *Enterobacterales* included in the screening for mobile genetic elements mediating antimicrobial resistance. Further details are provided elsewhere [56].

Species and Strain Number (73)	Sequence Type	Acquired Antimicrobial Resistance Genes	Recorded Phenotypic Resistance Against Apart From Penicillins and Cephalosporines *	Short-Read Archive (SRA) Accession Number
*Klebsiella pneumoniae* (73)	ST39	*bla*_TEM-1B_, *bla*_CTX-M-15,_*sul1*, *fosA*, *dfrA27*, *erm(B)*, *mph(A)*, *tet(D)*, *oqxB*, *oqxA*, *aac(6′)-Ib-cr*, *qnrB2*, *catA2*-like, *aadA16*, *aac(3)-IIa*, *aph(3″)-Ib*, *aph(6)-Id*	gentamicin, ciprofloxacin, moxifloxacin, trimethoprim/sulfamethoxazole	SRR13617236
*Klebsiella pneumoniae* (100)	ST152	*bla*_CTX-M-15_, *bla*_OXA-1_, *bla*_TEM-1B,_*sul2*, *sul1*, *dfrA1*, *dfrA27*, *mph(A)*, *aac(6′)-Ib-cr*, *oqxB*, *qnrB6*, *oqxA*, *catB3*, *catA1*, *ARR-3*, *aac(3)-IIa*, *aph(6)-Id*, *aph(3″)-Ib*, *aadA1*, *aadA16*, *aph(3′)-Ia*	gentamicin, ciprofloxacin, moxifloxacin, trimethoprim/sulfamethoxazole	SRR13617311
*Klebsiella pneumoniae* (199)	ST17	*bla*_CTX-M-15_, *bla*_TEM-1B,_*sul2*, *sul1*, *fosA*-like, *dfrA16*, *oqxA*, *oqxB*, *aadA2b*, *aac(3)-IIa*	gentamicin, trimethoprim/sulfamethoxazole	SRR13617280
*Klebsiella pneumoniae* (267)	ST36	*bla*_CTX-M-15_, *bla*_TEM-1B,_*sul2*, *sul1*, *fosA*, *dfrA27*, *tet(D)*, *aac(6′)-Ib-cr*, *oqxA*, *oqxB*, *catA2*-like, *ARR-3*, *aph(6)-Id*, *aph(3″)-Ib*, *aadA16*, *aac(3)-IIa*	gentamicin, trimethoprim/sulfamethoxazole	SRR13617257
*Escherichia coli* (41)	ST2	*bla*_OXA-1_, *bla*_TEM-1B_, *bla*_CTX-M-15,_*sul1*, *dfrA17*, *mph(A)*, *tet(B)*, *aac(6′)-Ib-cr*, *catB3*, *catA1*, *aac(3)-IId*, *aadA5*, *mdf(A)*	gentamicin, ciprofloxacin, moxifloxacin, trimethoprim/sulfamethoxazole	SRR13617294
*Escherichia coli* (225)	ST506	*bla*_TEM-1D_, *bla*_CTX-M-15,_*sul1*, *sul2*, *dfrA17*, *mph(A)*, *tet(A)*, *catA1*, *aadA5*, *aph(6)-Id*, *aph(3″)-Ib*, *mdf(A)*-like	moxifloxacin, trimethoprim/sulfamethoxazole	SRR13617270
*Escherichia coli* (270)	ST2	*bla*_CTX-M-15_, *tet(B)*, *catA1*, *mdf(A)*	ciprofloxacin, moxifloxacin	SRR13617256

* Only resistance according to EUCAST is recorded, while intermediate susceptibility has been attributed to the susceptibility group. All strains were phenotypically resistant against ampicillin, ampicillin/sulbactam, piperacillin/tazobactam, cefuroxime, cefuroxime axetil, cefpodoxime, cefotaxime and ceftazidime. Other tested antimicrobial drugs comprised ertapenem, imipenem, meropenem, gentamicin, ciprofloxacin, moxifloxacin, tigecycline and trimethoprim/sulfamethoxazole.
